# Nuclear Pyruvate Kinase M2 (PKM2) Contributes to Phosphoserine Aminotransferase 1 (PSAT1)-Mediated Cell Migration in EGFR-Activated Lung Cancer Cells

**DOI:** 10.3390/cancers13163938

**Published:** 2021-08-04

**Authors:** Rumeysa Biyik-Sit, Traci Kruer, Susan Dougherty, James A. Bradley, Daniel W. Wilkey, Michael L. Merchant, John O. Trent, Brian F. Clem

**Affiliations:** 1 Department of Biochemistry and Molecular Genetics, University of Louisville School of Medicine, Louisville, KY 40202, USA; rumeysa.biyiksit@louisville.edu (R.B.-S.); traci.kruer@moffitt.org (T.K.); susan.dougherty@louisville.edu (S.D.); james.bradley@louisville.edu (J.A.B.); 2 Department of Medicine, Division of Nephrology and Hypertension, University of Louisville School of Medicine, Louisville, KY 40202, USA; Daniel.wilkey@louisville.edu (D.W.W.); Michael.merchant@louisville.edu (M.L.M.); 3 Department of Medicine, Division of Hematology and Oncology, University of Louisville School of Medicine, Louisville, KY 40202, USA; john.trent@louisville.edu; 4 Brown Cancer Center, University of Louisville School of Medicine, Louisville, KY 40202, USA

**Keywords:** NSCLC, EGFR mutation, motility, phosphoserine aminotransferase 1, pyruvate kinase

## Abstract

**Simple Summary:**

Alternative functions for metabolic proteins have recently been shown to drive cancer growth. These may include differential enzymatic activity or novel protein associations. Phosphoserine aminotransferase 1 (PSAT1) participates in cellular serine synthesis and has been observed to be elevated in different tumor types. In this study, we aimed to identify new putative PSAT1 activities and determine their contribution to lung tumor progression. We found a direct association for PSAT1 with another enzyme, pyruvate kinase M2. While this appears not to affect PKM2’s metabolic activity, PSAT1 is required for the specific cellular localization of PKM2 upon tumorigenic signaling. Further, the depletion of PSAT1 suppresses lung cancer cell movement that can be partially restored by the compartment expression of PKM2. These findings reveal a novel mechanism that is able to promote the spread of this deadly disease.

**Abstract:**

An elevated expression of phosphoserine aminotransferase 1 (PSAT1) has been observed in multiple tumor types and is associated with poorer clinical outcomes. Although PSAT1 is postulated to promote tumor growth through its enzymatic function within the serine synthesis pathway (SSP), its role in cancer progression has not been fully characterized. Here, we explore a putative non-canonical function of PSAT1 that contributes to lung tumor progression. Biochemical studies found that PSAT1 selectively interacts with pyruvate kinase M2 (PKM2). Amino acid mutations within a PKM2-unique region significantly reduced this interaction. While PSAT1 loss had no effect on cellular pyruvate kinase activity and PKM2 expression in non-small-cell lung cancer (NSCLC) cells, fractionation studies demonstrated that the silencing of PSAT1 in epidermal growth factor receptor (EGFR)-mutant PC9 or EGF-stimulated A549 cells decreased PKM2 nuclear translocation. Further, PSAT1 suppression abrogated cell migration in these two cell types whereas PSAT1 restoration or overexpression induced cell migration along with an elevated nuclear PKM2 expression. Lastly, the nuclear re-expression of the acetyl-mimetic mutant of PKM2 (K433Q), but not the wild-type, partially restored cell migration in PSAT1-silenced cells. Therefore, we conclude that, in response to EGFR activation, PSAT1 contributes to lung cancer cell migration, in part, by promoting nuclear PKM2 translocation.

## 1. Introduction

The impact of metabolic reprogramming has been well-accepted in cancer pathogenesis [1,2]. The contribution of the Warburg effect and glutamine addiction has mainly been extensively investigated in the growth and survival of multiple tumor types [3,4,5]. More recently, several reports have described additional functions for glycolytic enzymes in tumor progression beyond their metabolic activities [6]. For example, PKM2 can be translocated into the nucleus in response to a variety of oncogenic signals and regulate gene expression, particularly through a direct interaction with transcription factors or transcription factor phosphorylation by inherent protein kinase activity [7,8,9,10,11]. Through the interaction with ACTA2, phosphoglycerate mutase 1 (PGAM1) mediates the actin filament assembly and increases cell migration and invasion in breast cancer [12]. Although the pro-tumorigenic functions of glycolytic enzymes are well-established, the requirement for serine synthesis enzymes has only recently been described [13,14,15,16,17].

The *de novo* cellular production of serine may contribute to tumor growth by providing precursors for macromolecular production and one-carbon metabolism [17]. Accordingly, multiple cancers exhibit an increased expression of serine synthesis pathway (SSP) enzymes [15,16,18,19]. For example, elevated PSAT1, which catalyzes the second step in converting 3-phosphohydroxypyruvate to phosphoserine, is associated with poorer clinical outcomes [14,20,21,22,23,24,25,26,27]. Depending on the tumor type, several reports have implicated PSAT1 in many oncogenic processes including proliferation, migration, invasion, and chemo-resistance [14,19,20,21,24,25,28,29]. However, the complete mechanism by which the serine biosynthetic pathway facilitates metabolic or cellular changes necessary for tumor growth is still not fully understood [17]. As observed with certain glycolytic proteins, studies have also described the non-canonical activities of SSP enzymes. Phosphoglycerate dehydrogenase (PHGDH) contributes to glioma progression through a direct interaction and stabilization of FOXM1 [16]. Separately, phosphoserine phosphatase (PSPH) can promote tumorigenesis through direct IRS1 dephosphorylation [15]. While multiple studies indicate a pro-tumorigenic role for PSAT1, alternative functions for this SSP enzyme have yet to be fully described.

We now report a novel direct interaction between PSAT1 and PKM2. This association was confirmed in two NSCLC cell types but the loss of PSAT1 did not alter the cellular PKM2 expression or activity. We further found that both PSAT1 and PKM2 exhibited a nuclear translocation in response to EGFR activation in lung cancer cells whereas PSAT1 suppression abrogated the PKM2 nuclear localization. PSAT1 silencing decreased the migration in these cell types but PSAT1 restoration or overexpression promoted cell motility and PKM2 nuclear localization. Lastly, the re-expression of a nuclear localization signal (NLS)-tagged PKM2 acetyl-mimetic (K433Q) mutant partially restored cell migration in PSAT1-suppressed cells. Taken together, our findings suggest that nuclear PKM2 translocation contributes, in part, to PSAT1-mediated cell migration under EGFR activation in lung cancer.

## 2. Materials and Methods

### 2.1. Reagents and Antibodies

Erlotinib was purchased from Selleckchem (OSI-744, Houston, TX, USA). Human recombinant proteins PKM2 (SAE0021), PKM1 (SRP0415), and EGF (E9644); an anti-β-actin (A2228) antibody; PSAT1 shRNA (TRCN0000291729) and control shRNA (SHC202) were purchased from Sigma-Aldrich (St. Louis, MO, USA). Antibodies against PKM2 (4053), PKM1 (7067), Oct-1 (8157), 𝑎-tubulin (3873), DYKDDDDK-Tag (2368) and rabbit IgG (2729) were obtained from Cell Signaling Technology (Danvers, MA, USA). An anti-PSAT1 (10501-1-AP) antibody was purchased from Proteintech Group Inc. (Rosemont, IL, USA). The PSAT1 Double Nickase CRISPR Plasmid system (sc-403001-NIC) was purchased from Santa Cruz Biotechnology (Dallas, TX, USA).

### 2.2. GST-Pulldown and Mass Spectrometry

PSAT1 cDNA was subcloned into the pGEX4T-1 plasmid (GE Healthcare, Chicago, IL, USA) to generate pGEX-GST-PSAT1 and tagged PSAT1 was induced in BL21 cells with IPTG. GST-PSAT1 was incubated with one milligram of pre-cleared A549 lysate. Columns were subsequently washed 3X and eluted with reduced glutathione. Dialyzed elutes were separated by SDS-PAGE and protein bands were detected by a silver stain. Bands enriched in the GST-PSAT1 samples were excised and sent for protein identification by mass spectrometry.

### 2.3. LC/MS Data Collection and Analysis

Tryptic peptides were prepared from excised gel bands and analyzed using a liquid chromatograph-tandem mass spectrometry (LC-MS/MS) approach as previously described [30]. Briefly, peptides were separated on a 10 cm C-18 (Jupiter 5 µm RP300A Phenomenex) packed needle tip using a 5% to 40% acetonitrile gradient at a flow rate of 200 nL/min prior to nanoelectrospray into an LTQ linear ion trap mass spectrometer (Thermo Fisher Scientific, Waltham, MA, USA). Data were acquired in a data-dependent fashion with a full MS scan (300–2000 m/z) followed by six MS/MS scans (35% collision energy) and a 1 min dynamic exclusion window. MS/MS data were searched by ProteomeDiscoverer (version 1.4; Thermo Fisher Scientific, Waltham, MA, USA) as previously described against a human refseq protein database (version HumanRef140722.fasta with 88,942 entries) using SEQUEST (version 1.4.0.288; Thermo Fisher Scientific, Waltham, MA, USA) and Mascot algorithms (version 2.4; Matrix Science, Boston, MA, USA) assuming a 1.0 Da fragment mass tolerance and a 1.2 Da parent mass tolerance, a fixed modification of cysteine (+57 for carbamidomethylation), a variable oxidation of methionine (+ 16to methionine) and maximal two missed trypsin cleavages [31]. High-probability peptide and protein identifications were assigned using the Peptide-/Protein-Prophet algorithms (http://tools.proteomecenter.org/software.php; accessed on 21 August 2014) and quantitated using Scaffold v4.3.4 (ProteomeSoftware, Portland, OR, USA) using a spectral counting method.

### 2.4. Cell Culture

A549 human lung cancer cells (ATCC) and HEK293T human embryonic kidney cells (provided by Dr. Geoffrey Clark after STR profiling) were maintained in DMEM (Gibco, Grand Island, NY, USA) supplemented with 10% FBS and 50 µg/mL gentamicin (Gibco). PC9 human lung cancer cells (provided by Dr. Levi Beverly after STR profiling) were maintained in RPMI media (Gibco) supplemented with 10% FBS and 50 µg/mL gentamicin (Gibco). All cells were cultured in humidified incubators at 37 °C and 5% CO_2_.

### 2.5. Plasmid Constructions for Wild-Type and Mutant PKM2

Full-length human PKM2 cDNA was amplified from A549 cells using primers forward: 5′-CTGGGGATCCATGTCGAAGCCCCATAGTGAAG-3′ and reverse: 5′-GATCGAATTCTCACGGCACAGGAACAACACGC-3′ and subcloned into the pcDNA 3.1/FLAG vector (FLAG-PKM2 wild-type (WT)). FLAG-PKM2 WT plasmid was used to generate mutant PKM2 expression constructs using either a QuikChange site-directed mutagenesis kit (Agilent, Santa Clara, CA, USA) or a Q5 site-directed mutagenesis kit (New England Biolabs, Ipswich, MA, USA). The primers utilized for the PKM2 site-directed mutagenesis are listed in Table S1. All plasmid constructs were verified by a sequencing analysis (Eurofins Genomics, Louisville, KY, USA).

### 2.6. Transient Transfection

HEK293T cells were transfected with either FLAG-tagged empty vector (FLAG-EV) (FLAG-HA-pcDNA3.1 (Addgene, # 52535, Watertown, MA, USA)), PKM2 WT or mutant PKM2 (MT(1–4)) vectors using jetPEI (PolyPlus, New York, NY, USA) according to the manufacturer’s protocol. Forty-eight hours post-transfection, the cells were lysed in an IP lysis buffer (Pierce, Rockford, IL, USA) and co-immunoprecipitation was performed.

### 2.7. Generation of Stable Cell Lines

PSAT1 stable knockdown: A549 or PC9 cells were transfected with shRNA (PSAT1 shRNA or non-targeted mammalian control shRNA) using jetPEI and clonal cells were selected in 1 µg/mL puromycin (Sigma, St., MO, USA).

PSAT1 genetic knockout: PC9 cells were transfected with the PSAT1 CRISPR/Cas9n(D10A)-Puromycin nickase plasmid using jetPEI. Stably transfected cells were selected in 1 µg/mL puromycin. To achieve PSAT1 deletion, puromycin-selected PC9 cells were transfected with the PSAT1 CRISPR/Cas9n(D10A)-GFP nickase plasmid. PSAT1 knockout cells were clonally expanded from GFP-positive cells and validated by immunoblotting. Puromycin-selected cells without GFP nickase transfection served as control knockout cells.

Ectopic FLAG-tagged PSAT1 expression: PSAT1 cDNA (forward: 5′-TGGGATCCATGGACGCCCCCAGGCAGGTG-3′ and reverse: 5′-TGGAATTCTCATAGCTGATGCATCTCCAA-3′) was cloned into pcDNA 3.1/FLAG vector. PC9 cells were transfected with FLAG-tagged PSAT1 or FLAG-EV using jetPEI and selected in 200 µg/mL geneticin (Gibco).

PSAT1 rescue studies: An shRNA-resistant FLAG-tagged PSAT1 expression plasmid was generated by site-directed mutagenesis (the primers are listed in Table S1). For these studies, shRNA-resistant FLAG-tagged PSAT1 was co-transfected with PSAT1 shRNA into parental PC9 cells. Alternatively, PC9 cells were co-transfected with either FLAG-EV and non-targeted shRNA or PSAT1 shRNA to serve as a control and PSAT1-silenced PC9 cells, respectively. Stably transfected cells were selected in 200 µg/mL geneticin (Gibco) and 1 µg/mL puromycin. 

Nuclear PKM2 overexpression: PKM2 cDNA (forward: 5′TATTTAGGCGCGCCATGTCGAAGCCCCATAGTGAAG-3′ and reverse: 5′-GCCCGTTAATTAATCACGGCACAGGAACAACACGC-3′) was subcloned into a pcDNA-3xFLAG-NLS vector (Addgene, #53585). A FLAG-PKM2^NLS-K433Q^ expression plasmid was generated by site-directed mutagenesis (the primers are listed in Table S1). All constructs were verified by a sequencing analysis. shPSAT1 PC9 cells were transfected with FLAG-PKM2^NLS-WT^, FLAG-PKM2^NLS-K433Q^ or FLAG-EV using jetPEI. Control PC9 cells were transfected with FLAG-EV only. Stably transfected cells were selected in 200 µg/mL geneticin and 1 µg/mL puromycin.

### 2.8. EGF and Erlotinib Treatment

EGF treatment: Stable A549 control and shPSAT1 cells were serum-starved in serum-free DMEM for 24 h and subsequently treated with 100 ng/mL EGF or a vehicle (10 mM acetic acid) for 6 h. The cells were then collected for subcellular fractionation.

Erlotinib treatment: Stable PC9 control and shPSAT1 cells were treated with 1 µM erlotinib or a vehicle (DMSO) in serum-free RPMI media for 48 h prior to a subcellular fractionation analysis.

### 2.9. Subcellular Fractionation

Cytosolic and nuclear proteins were isolated using an NE-PER kit (Thermo Scientific, 78835, Rockford, IL, USA). A total of 15 µg of cytoplasmic protein and 25 µg of nuclear protein were used for the immunoblotting analyses.

### 2.10. Co-Immunoprecipitation (Co-IP)

One milligram of cell lysate was incubated with one microgram of either an anti-PSAT1 or an anti-rabbit IgG antibody. Immunoprecipitates were collected using Protein G Dynabeads (Invitrogen, 10004D, Carlsbad, CA, USA) and immunoprecipitated proteins were analyzed by immunoblot.

For recombinant Co-IPs, 50 ng of recPSAT1 protein was mixed with 500 ng of recPKM1 or recPKM2. RecPSAT1 in an IP lysis buffer alone was used as a negative control.

### 2.11. Immunoblotting

Protein extracts or co-immunoprecipitations were separated by SDS-PAGE and transferred to the PVDF membrane. Blocked membranes were then incubated with the indicated primary antibodies. The protein detection was performed using HRP-conjugated secondary antibodies and visualized by chemiluminescence.

### 2.12. Immunofluorescence Staining

Cells were plated in 4-well chamber slides, incubated in serum-free media for 24 h and fixed in 4% paraformaldehyde. After blocking, the cells were incubated with primary anti-PKM2 and Alexa Fluor 488-conjugated goat anti-rabbit secondary antibodies (Invitrogen, A-11034). The images were captured by an Olympus FV-3000 confocal microscope equipped with Fluoview software under 40 × magnification.

### 2.13. Wound Healing Assay

All PC9 cell lines were plated at 10^6^ cells/well in a 6-well plate and grown in complete media overnight. Experimental conditions for motility assays were used to minimize the contribution of cell proliferation on wound healing as described previously [32]. After plating, the cells were incubated in serum-free media for 24 h. The confluent monolayer of cells was then wounded with a pipette tip, washed three times and cultured in low-serum RPMI media (1%). The images were captured at 0 and 24 h and analyzed using Image J software with an MRI wound healing tool [33]. The migrated area was calculated by the subtraction of the wound area (arbitrary unit) at 24 h from the initial wound area.

### 2.14. Transwell Migration Assay

A549 control and shPSAT1 stable cell lines were plated at 70% confluency and cultured in complete media for 24 h. The cells were then serum-starved for 24 h and 10^5^ cells were plated in serum-free media in a Boyden chamber. Serum-free media supplemented with 100 ng/mL EGF or a vehicle (10 mM acetic acid) was added to the bottom chamber. After 24 h, the cells were fixed with 100% ice-cold methanol and the non-migrated cells were removed. The migratory cells were stained with 0.05% crystal violet and washed with ddH_2_O. The area of stained cells was quantified using the Image J threshold tool as described [34].

### 2.15. Pyruvate Kinase Activity Assay

Both A549 and PC9 control and shPSAT1 stable cells were cultured to 70% confluency in a 6-well plate. Intracellular pyruvate kinase activity was assessed using the Pyruvate Kinase Activity assay kit (Sigma, MAK072) according to the manufacturer’s protocol.

### 2.16. Computational Homology Modeling of PKM1 and PKM2

The human PKM1 sequence was obtained from the PKM1 crystal structure (Protein Data Bank entry 3SRF) and was mapped to the human PKM2 structure (Protein Data Bank entry 4FXJ) using the Prime-Based Homology Modeling Module of the Schrödinger Software Suite [35]. The image was created within Schrödinger Maestro with the sequence differences between PKM1 and PKM2 identified.

### 2.17. Bioinformatic Analysis of PSAT1 in EGFR-Mutant Lung Cancers

The GSE32863, GSE75037, GSE31548, GSE31210 and GSE11969 datasets encompassing the gene expression analysis and correlating the clinical information from EGFR-mutant lung cancers were downloaded from the Gene Expression Omnibus (GEO) database using BRB-ArrayTool software [36]. The *PSAT1* expression (probe IDs: ILMN_1692938 for GSE32863 and GSE75037, 223062_s_at for GSE31210 and GSE31548 and 5144 for GSE11969) was retrieved from EGFR-mutant tumor and normal lung specimens. Differential expression analyses were carried out for tumor vs. normal and stage II–III vs. stage I while Kaplan–Meier analyses for the overall survival and relapse-free survival rates were performed on the indicated datasets.

### 2.18. Statistical Analysis

All data were statistically analyzed using GraphPad Prism 8 software (Version 8.4, San Diego, CA, USA. Statistical significances were assessed based on the number of groups with one or more independent variables. Paired t-tests were used for the analysis of two groups, with the exception of an unpaired t-test for the pyruvate kinase assay. While a one-way ANOVA with Tukey’s multiple comparison test for three groups was performed for PC9 cell variants, a two-way ANOVA with Tukey’s multiple comparison test was used for A549 cell variants with or without EGF treatment. The Prism-Survival analysis tool was utilized to obtain the overall survival (OS) and relapse-free survival (RFS) plots. Experimental replicates for each analysis are stated within the respective figure legend. Values of *p* < 0.05 were considered statistically significant.

## 3. Results

### 3.1. PKM2 Is a Novel Binding Partner of PSAT1

**Figure 1:**
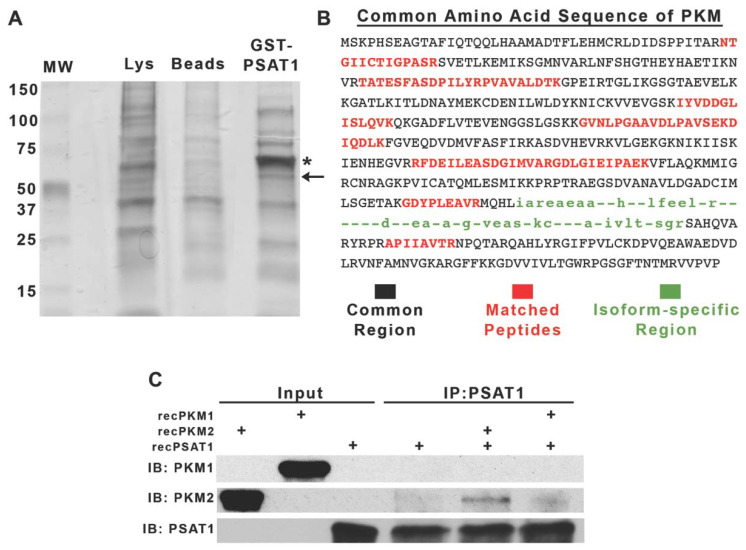
PKM2 is a novel binding partner of PSAT1. (**A**) Silver stain of GST-PSAT1-purified proteins from A549 whole-cell lysates. * Denotes residual GST-PSAT1 from column purification; ← denotes gel slice encompassing PKM. (**B**) Primary amino acid sequence of human PKM. MS-identified peptides of PKM are highlighted in red. Black-labeled sequences belong to common regions of both PKM1 and PKM2 isoforms and green-labeled sequences identify isoform specificity. (**C**) Co-IP of recombinant (rec-) PSAT1 and PKM1 or PKM2. Immunocomplexes were precipitated using an anti-PSAT1 antibody and analyzed by immunoblot using anti-PKM1, anti-PKM2, and anti-PSAT1 antibodies. Recombinant proteins were used as input controls showing antibody specificity and PSAT1 alone was used as an IP control. Shown are representative images from two separate experiments.

The non-canonical functions of metabolic enzymes that directly involve protein–protein interactions have previously been reported in promoting tumorigenesis [37,38]. To determine PSAT1-associating proteins, we used a GST-PSAT1 pulldown assay coupled to an LC-MS/MS analysis (Figure 1A). Among the resolved proteins, we identified pyruvate kinase M (PKM) with approximately a 22% sequence coverage (Figure 1B). PKM1 and PKM2 are two alternatively spliced isoforms of the *PKM* gene and exhibit differential enzymatic properties [39]. Utilizing a co-IP analysis encompassing only recombinant PKM1, PKM2, and PSAT1, we found that PSAT1 directly binds to the PKM2 isoform (Figure 1C). Thus, these results identify PKM2 as a novel direct binding partner of PSAT1.

**Figure 2:**
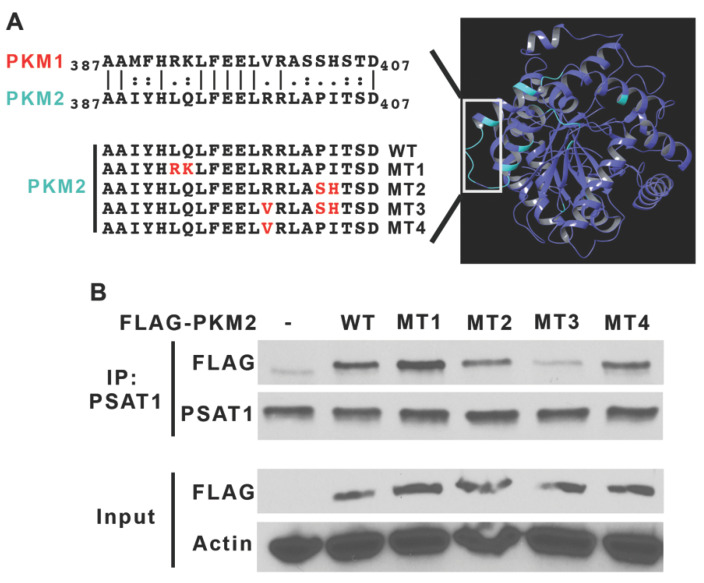
Mutations within an isoform-specific region of PKM2 weakens the PSAT1 interaction. (**A**) Schematic representation of PKM2-specific mutations generated for the analysis of the PSAT1–PKM2 association. Ribbon representation of the structure of PKM2 colored by PKM1 sequence homology (right panel). Identical regions are shown in purple and divergent regions in cyan. The left panel depicts the site-directed mutagenesis of amino acids (MT1–4, highlighted in red) in the PKM2-specific region (denoted in the white box). (**B**) FLAG-PKM2 wild-type (WT), mutants (MT1–4) and FLAG-EV (-) were expressed in HEK293T cells. Endogenous PSAT1 protein complexes were immunoprecipitated and the association with PKM2 was assessed by immunoblot. A similar expression of FLAG-PKM2 variants is shown by immunoblot of FLAG fusion proteins from the protein lysate input with β-actin used for a protein loading control. Shown are representative images from three separate experiments. (-) denotes an empty vector.

Based on this interaction, we hypothesized that a PKM2-specific region may contribute to a PSAT1 association. Three-dimensional modeling of human PKM1 and PKM2 demonstrates that they have a 95.8% sequence homology and have the same overall structure. As depicted in Figure 2A, an isoform-specific region (cyan) is localized to a loop structure within the respective monomers. We mutated amino acid residues in wild-type (WT) PKM2 to the corresponding PKM1 residues by site-directed mutagenesis (Figure 2A, mutations highlighted in red). A Co-IP analysis involving a FLAG-tagged PKM2 WT and mutant (MT 1–4) proteins revealed that mutations in MT 1, 2 and 4 did not substantially affect the interaction with PSAT1 (Figure 2B). However, the amino acid changes within MT3-PKM2 that are inclusive of mutations in both MT 2 and 4 strongly reduced the binding to endogenous PSAT1. Together, the recombinant co-IP and mutational analysis demonstrate a specific PSAT1–PKM2 interaction.

### 3.2. Suppression of PSAT1 in NSCLC Cancer Cells Does Not Alter PKM2 Expression or Pyruvate Kinase Activity

**Figure 3:**
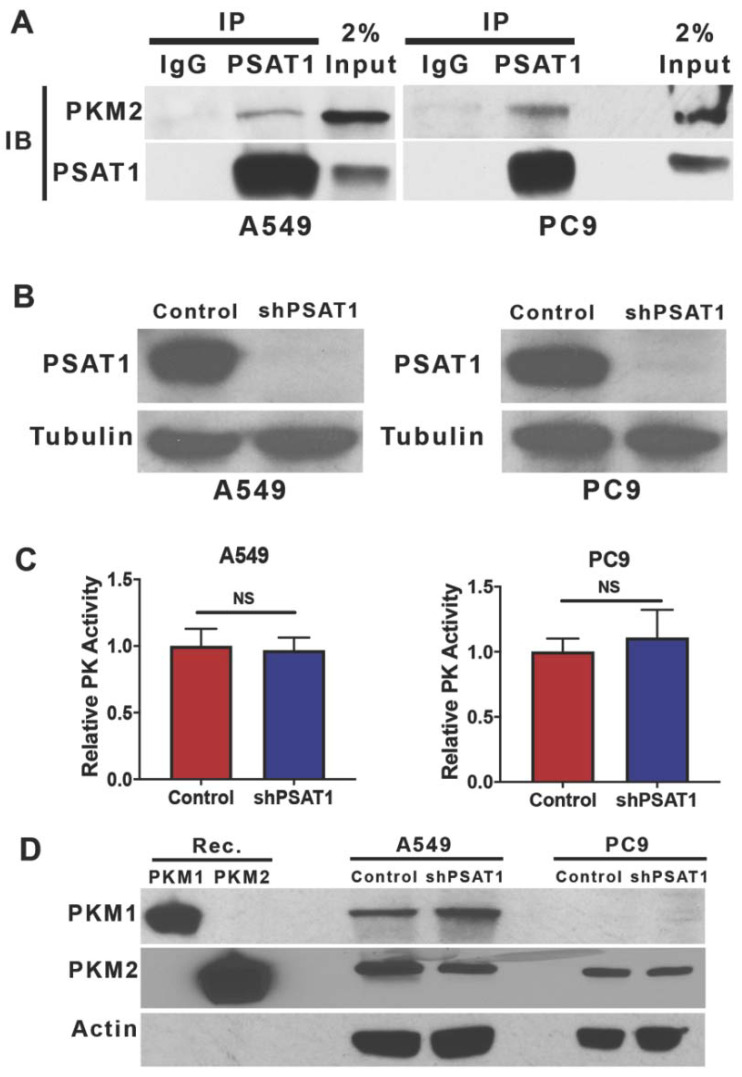
PSAT1 associates with endogenous PKM2 in NSCLC cells but the loss of PSAT1 does not alter either PKM2 expression or pyruvate kinase activity. (**A**) Co-IP of PSAT1 and PKM2 in A549 and PC9 NSCLC cells. PSAT1-specific immunocomplexes were precipitated using anti-PSAT1 from a whole-cell lysate and analyzed for PKM2 by immunoblot with an anti-PKM2 antibody. Shown are representative images from three separate experiments. (**B**) Loss of PSAT1 expression in A549 and PC9 cells stably expressing PSAT1-specific shRNA. The PSAT1 expression was determined in whole-cell lysates from the control or shPSAT1-expressing cells by immunoblot using anti-PSAT1 and anti-α-tubulin (loading control). (**C**) Intracellular pyruvate kinase activity was determined in cell lysates from A549 or PC9 cells with or without PSAT1 expression. Data are represented as relative pyruvate kinase (PK) activity (control cells set to 1) and shown are the mean ± SD from four independent experiments. A statistical significance was determined by an unpaired t-test analysis. (**D**) Immunoblot analysis of PKM1 or PKM2 expression in whole-cell lysates from the control or PSAT1-silenced A549 and PC9 cells. Recombinant human PKM1 and PKM2 proteins were used as positive controls for antibody specificity and β-actin was used as a loading control. Shown are representative images from two separate experiments. IP: immunoprecipitation; IB: immunoblot; NS: not significant.

Previously, the pyruvate kinase activity of PKM2 has been metabolically linked to the serine biosynthetic pathway in human cancers [40,41,42,43]. Our pulldown and recombinant protein association studies now suggest that these pathways might also be connected via protein–protein interactions in tumor cells. A Co-IP analysis demonstrated an endogenous interaction between PSAT1 and PKM2 in two separate NSCLC cell systems, *KRAS*-mutant A549 cells and *EGFR*-mutant PC9 cells (Figure 3A).

To investigate the functional significance of this PSAT1 and PKM2 interaction, the PSAT1 expression was stably silenced in both the A549 and PC9 cells (Figure 3B). We questioned whether the loss of PSAT1 may modulate the metabolic activity of PKM2. As shown in Figure 3C, PSAT1 depletion did not affect pyruvate kinase activity in either cell line. This was not due to an altered PKM2 or PKM1 expression as an analysis of the PKM isoform expression found no significant change between the control and shPSAT1 cells in both A549 and PC9 cells (Figure 3D). Taken together, we conclude that while a physiological interaction between PSAT1 and PKM2 exists, the suppression of PSAT1 expression fails to alter pyruvate kinase activity and the expression of PKM2 in these NSCLC cell lines indicating that this interaction may be dispensable for cellular pyruvate kinase activity.

### 3.3. Silencing of PSAT1 Suppresses the Nuclear Localization of PKM2 in EGFR-Activated NSCLC Cells

**Figure 4:**
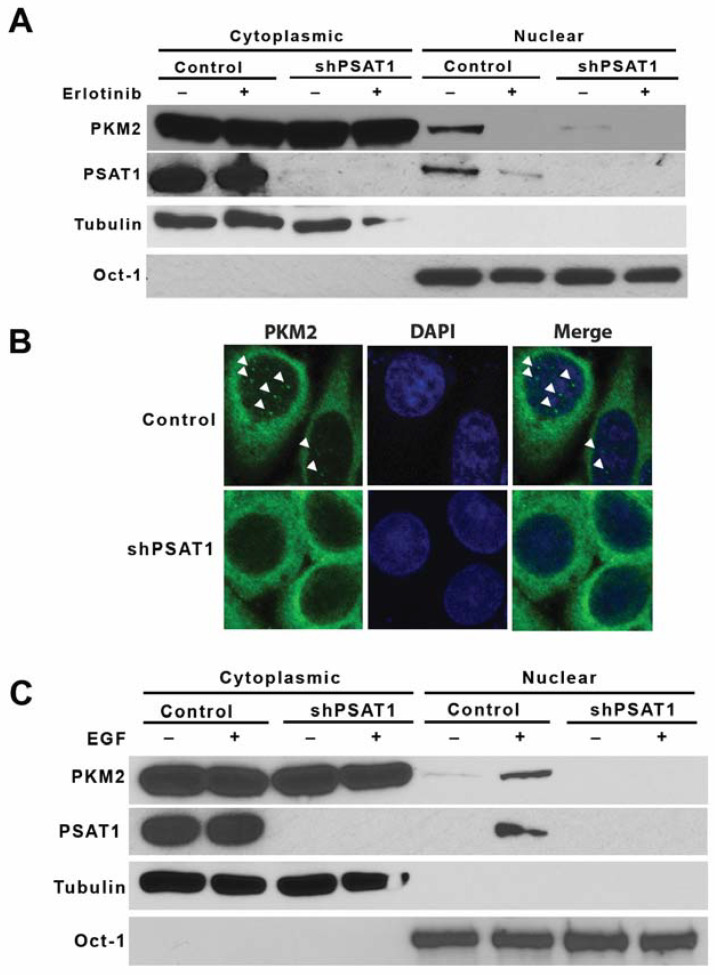
Silencing of PSAT1 suppresses the nuclear localization of PKM2 in EGFR activated NSCLC cells. (**A**) EGFR-mutant PC9 cells stably expressing the control or PSAT1 shRNA were treated with 1 µM of erlotinib. Cytoplasmic and nuclear fractions were examined by an immunoblot analysis using anti-PKM2 and anti-PSAT1 antibodies. Oct-1 and α-tubulin served as loading controls for the nuclear and cytoplasmic compartments, respectively. Shown are representative images from three separate experiments. (**B**) Nuclear localization of PKM2 was examined in serum-starved PC9 cells expressing the control or PSAT1 shRNA by confocal microscopy. DAPI served as a control for nuclear staining. Arrowheads indicate nuclear PKM2 staining in representative images from three independent experiments. (**C**) Serum-starved A549 cells (EGFR wild-type) stably expressing control or PSAT1 shRNA were treated with or without EGF (100 ng/mL). Cytoplasmic and nuclear fractions were prepared and the PKM2 and PSAT1 localizations were analyzed by immunoblot. Oct-1 and α-tubulin served as loading controls for the nuclear and cytoplasmic compartments, respectively. Shown are representative images from three separate experiments.

As recent studies have demonstrated the EGFR-mediated nuclear localization of PKM2 [8,10,44], we examined whether PSAT1 may alter PKM2 nuclear translocation under EGFR activation in NSCLC cells. As PC9 cells exhibit constitutively active EGFR signaling due to the activating exon 19 deletion in the *EGFR* gene [45], it serves as a model to examine the role of PSAT1 in EGFR-mediated PKM2 nuclear localization. Subcellular fractionation confirmed the nuclear localization of PKM2 in PC9 cells that was blocked with the EGFR tyrosine kinase inhibitor, erlotinib (Figure 4A). Importantly, cellular fractionation and an immunofluorescence analysis found that the stable shRNA-mediated loss of PSAT1 diminished the PKM2 translocation under EGFR activation (Figure 4A,B). To validate this finding, the PSAT1 expression was depleted in PC9 cells via a CRISPR-mediated gene knockout and, consistent with the knockdown study, PSAT1 deletion abolished the nuclear localization of PKM2 in PC9 cells (Figure S1A). To complement these loss of function studies, FLAG-tagged PSAT1 was overexpressed in PC9 cells to examine the correlation between the level of PSAT1 expression and nuclear PKM2 (Figure S2A). A fractionation analysis showed that nuclear PKM2 is increased upon the ectopic expression of PSAT1 (Figure S2B). Unexpectedly, PSAT1 was also found within the nuclear compartment of PC9 cells, which was abrogated with erlotinib treatment and enhanced with a FLAG-tagged PSAT1 overexpression (Figure 4A, Figures S1A and S2B).

We further substantiated the effects of PSAT1 depletion in the A549 cell model, which harbors wild-type EGFR that is stimulated by EGF [46,47]. A fractionation analysis revealed an enhanced nuclear translocation of PKM2 in EGF-stimulated A549 cells, whereas the loss of PSAT1 significantly inhibited an induced PKM2 nuclear translocation (Figure 4C). Consistent with the findings in PC9 cells, the PSAT1 nuclear localization was also observed in EGF-stimulated A549 cells.

### 3.4. Loss of PSAT1 Suppresses the Migration of EGFR-Mutant and EGF-Induced EGFR-WT Lung Cancer Cells

**Figure 5:**
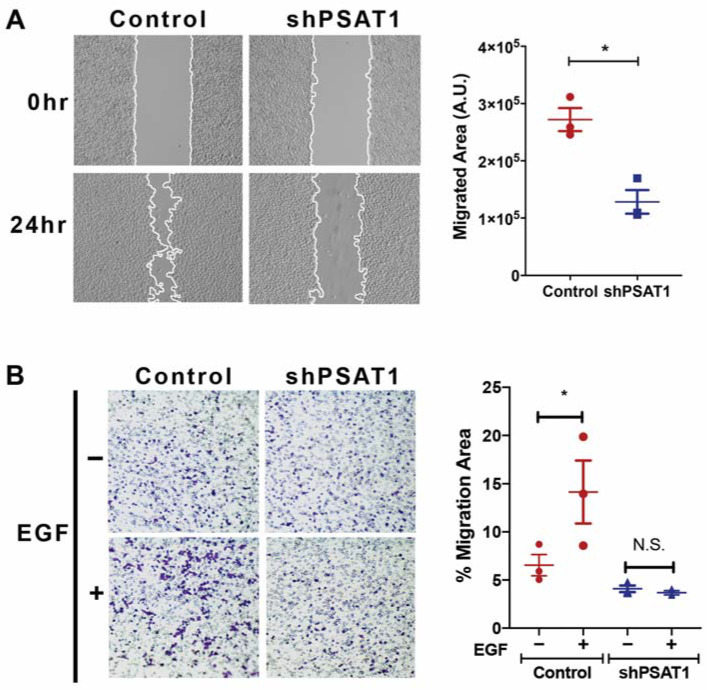
Loss of PSAT1 decreases cell migration in EGFR-mutant PC9 and EGF-stimulated A549 NSCLC cells. (**A**) Wound healing assay of PC9 cells expressing the control or PSAT1-specific shRNA. Shown are representative images taken at 0 h and 24 h. Migrating cells are demarcated by continuous white lines. Data are presented as a migrated area after 24 h and shown is the mean ± SE from three independent experiments. A statistical significance was determined by a paired t-test analysis. * is *p* < 0.005. (**B**) Boyden chamber migration assay on serum-starved A549 cells expressing the control or PSAT1 shRNA. A total of 100 ng/mL EGF serum-free media was used as a chemo-attractant and migrated cells were fixed and stained with crystal violet after 24 h. Shown are representative images of the migrated cells and quantification is demonstrated as the mean ± SE of the% migration area from three independent experiments. A statistical significance was determined by a two-way ANOVA with Tukey’s multiple comparison test. * *p* = 0.0001. N.S.: not significant; A.U.: arbitrary unit.

The oncogenic function of PSAT1 has been investigated in many tumor types and has been found to play a role in the proliferation, migration, and chemo-resistance [14,19,20,21,29,48,49] yet it remains elusive whether PSAT1 may contribute to the cellular response to specific oncogenic signaling, particularly EGFR. As previous studies have shown that EGF exposure selectively induces cell migration in A549 cells without affecting proliferation [46,47], we investigated the role of PSAT1 in cell motility in EGFR-activated NSCLC cells. We found that a loss of PSAT1, either via a shRNA-mediated knockdown or a CRISPR-mediated knockout, significantly decreased EGFR-mutant PC9 cell motility (Figure 5A and Figure S1B). To exclude the possibility of a proliferative defect on cell migration, we assessed cell proliferation under the experimental conditions and timing used to assess motility and observed no impact on cell proliferation with stable shRNA-mediated silencing (Figure S3). In addition, PSAT1 suppression completely inhibited EGF-induced cell migration in A549 cells (Figure 5B).

To extend these findings and investigate the clinical relevance of PSAT1 in EGFR-mutant lung cancer, we analyzed microarray datasets from patients with EGFR-mutant lung tumors for a comparative expression analysis and correlation with clinical outcomes. We found an elevated *PSAT1* expression in both paired and unpaired EGFR-mutant lung tumors compared with normal lungs (Figure S4A). Furthermore, late-stage EGFR-mutant lung tumors tended to exhibit an increased *PSAT1* expression compared with early-stage tumors, which is consistent with the elevated metastatic potential (Figure S4B). Finally, a Kaplan–Meier analysis correlated a high *PSAT1* expression with poorer relapse-free and overall survival rates in this patient population (Figure S4C). These findings suggest that a high PSAT1 expression negatively impacts the clinical outcomes in EGFR-mutant lung cancer.

**Figure 6:**
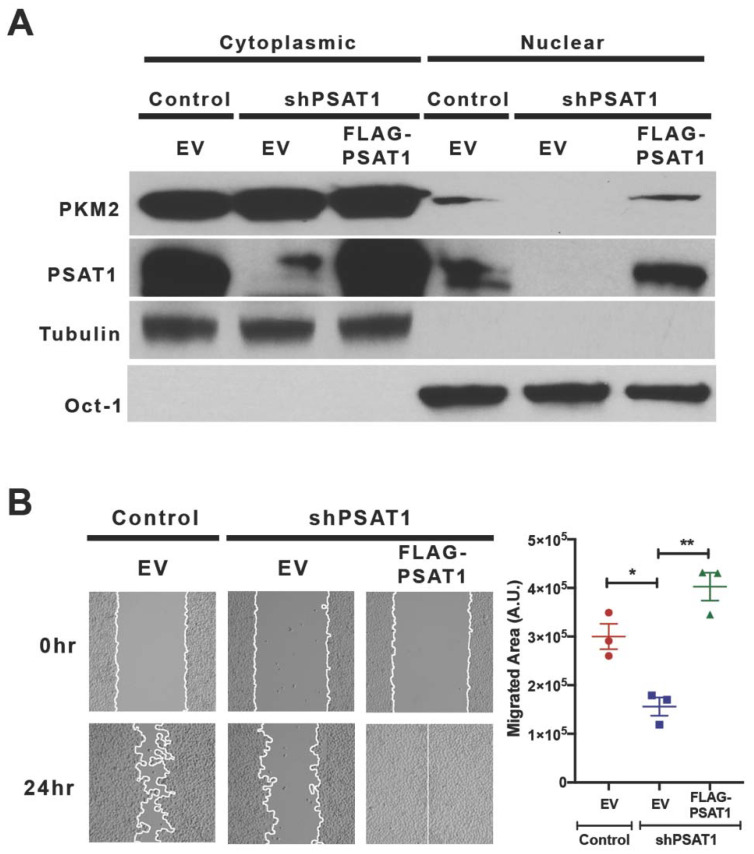
Re-expression of PSAT1 restores the nuclear localization of PKM2 and cell migration in silenced PC9 cells. (**A**) Immunoblot analysis for PKM2 and PSAT1 localization in PSAT1-silenced PC9 cells stably expressing an empty vector (EV) or FLAG-PSAT1. Cytoplasmic and nuclear fractions from the control-EV, shPSAT1-EV, and shPSAT1-FLAG-PSAT1 PC9 cells were analyzed using anti-PKM2 and anti-PSAT1 antibodies. Oct-1 and α-tubulin served as loading controls for the nuclear and cytoplasmic compartments, respectively. Shown are representative images from three independent experiments. (**B**) Wound healing assay of the control-EV, shPSAT1-EV, and shPSAT1-FLAG-PSAT1 PC9 cells. Shown are representative images at 0 h and 24 h. The migrating cells are demarcated by continuous white lines. Data are presented as a mean ± SE migrated area after 24 h from three independent experiments. A statistical significance was determined by a one-way ANOVA with Tukey’s multiple comparison test. ** *p* < 0.005 and * *p* < 0.05. A.U.: arbitrary unit.

To demonstrate PSAT1 specificity in our in vitro assays, we restored PSAT1 in silenced PC9 cells via the expression of shRNA-resistant FLAG-tagged PSAT1 (FLAG-PSAT1) (Figure 6A). The re-expression of PSAT1 rescued not only the nuclear localization of PKM2 but also the cell migration (Figure 6A,B and Figure S5). An increased cell migration upon an ectopic PSAT1 expression in parental cells further corroborates these findings (Figure S2C). These results demonstrate a role for PSAT1 in PKM2 nuclear localization and cell migration in these NSCLC models.

### 3.5. Re-Expression of Nuclear-Localized Acetyl-Mimetic (K433Q) PKM2 Partially Restores the Migration Defect Due to the Loss of PSAT1 in EGFR-Mutant Cells

**Figure 7:**
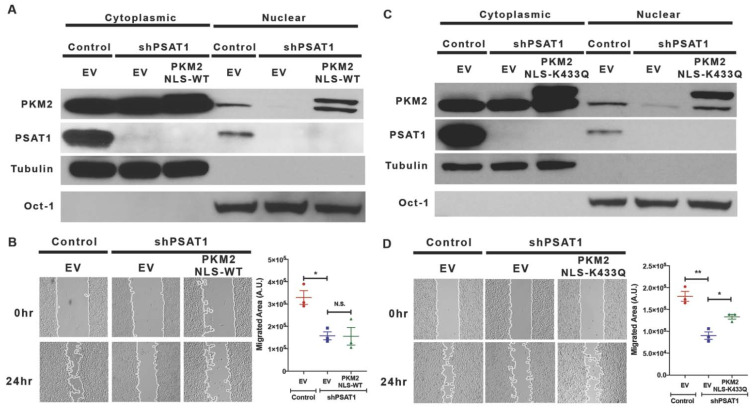
Re-expression of nuclear-localized acetyl-mimetic (K433Q) PKM2, but not wild-type PKM2, partially rescues the migration defect due to the loss of PSAT1. (**A**) Immunoblot analysis for PKM2 localization in PSAT1-suppressed PC9 cells stably expressing the nuclear-targeted wild-type PKM2 (FLAG-PKM2^NLS-WT^). Cytoplasmic and nuclear fractions from the control-EV, shPSAT1-EV, and shPSAT1-FLAG-PKM2^NLS-WT^-expressing cells were analyzed using anti-PKM2 and anti-PSAT1 antibodies. Oct-1 and α-tubulin served as loading controls for the nuclear and cytoplasmic compartments, respectively. Shown are representative images from three independent experiments. (**B**) Wound healing assay of serum-starved PC9 cells expressing the control-EV, shPSAT1-EV, or shPSAT1-FLAG-PKM2^NLS-WT^. Shown are representative images at 0 h and 24 h with migrating cells demarcated by continuous white lines. Data are presented as a mean ± SE migrated area after 24 h from three independent experiments. A statistical significance was determined by a one-way ANOVA with Tukey’s multiple comparison test. * *p* < 0.0001 and N.S.: not significant. (**C**) Immunoblot analysis for PKM2 localization in PSAT1-suppressed PC9 cells stably expressing nuclear-targeted acetyl-mimetic (K433Q) PKM2. Cytoplasmic and nuclear fractions from the control-EV, shPSAT1-EV, and shPSAT1-FLAG-PKM2^NLS-K433Q^-expressing cells were analyzed using anti-PKM2 and anti-PSAT1 antibodies. Oct-1 and α-tubulin served as loading controls for the nuclear and cytoplasmic compartments, respectively. Shown are representative images from three independent experiments. (**D**) Wound healing assay of serum-starved PC9 cells expressing the control-EV or shPSAT1-EV and shPSAT1-FLAG-PKM2^NLS-K433Q^. Shown are representative images at 0 h and 24 h with migrating cells demarcated by continuous white lines. Data are presented as a mean ± SE migrated area after 24 h from three independent experiments. A statistical significance was determined by a one-way ANOVA with Tukey’s multiple comparison test. ** *p* < 0.0001 and * *p* < 0.05. EV: empty vector; A.U.: arbitrary unit; NLS: nuclear localization signal.

As we observed a correlation between the nuclear PKM2 level and the cell migration rate upon altering the PSAT1 expression, we then assessed the requirement of nuclear PKM2 for PSAT1-mediated cell migration in EGFR-mutant cells. Nuclear PKM2 was reconstituted via the stable expression of FLAG-tagged PKM2 harboring a nuclear localization signal (FLAG-PKM2^NLS-WT^). However, the re-expression of nuclear PKM2 did not restore wound healing in PSAT1-depleted cells (Figure 7A,B).

Prior studies have revealed that nuclear PKM2 requires a specific post-translational modification to exert its nuclear function and found that oncogenic signals, including EGF, induce PKM2 acetylation at K433, which is found in the nucleus [7]. Furthermore, an acetyl-mimetic mutant (K433Q) form of PKM2 promoted tumor growth and metastasis in a xenograft model [7,50]. Consistent with this result, the SIRT6-mediated deacetylation of K433 results in the export of PKM2 from the nucleus and inhibits tumor growth and metastasis [50], suggesting that the acetylation of PKM2 at K433 is critical for nuclear localization and function. For this, we stably expressed acetyl-mimetic nuclear PKM2 (FLAG-PKM2^NLS-K433Q^) in cells lacking PSAT1. A fractionation analysis confirmed the reconstitution of acetyl-mimetic nuclear PKM2 (Figure 7C). Unlike wild-type PKM2, nuclear acetyl-mimetic PKM2 significantly rescued (41%) PC9 cell motility in the absence of PSAT1 (Figure 7D). Together, these results suggest that acetylated nuclear PKM2 contributes, in part, to PSAT1-mediated cell migration in EGFR-activated cells.

## 4. Discussion

An elevated PSAT1, along with other enzymes within the SSP, has previously been reported in lung cancer [13,14,51]. Notably, tumor-initiating cells display an increased expression of PSAT1, further emphasizing the functional significance of PSAT1 in lung cancer progression [52]. While the role of PSAT1 has been investigated in the context of metabolic function, the potential for an alternative role in tumorigenesis remains unclear. Recent discoveries of the multifunctionality of metabolic enzymes in tumorigenic processes led us to explore a putative non-canonical function of PSAT1 involved in lung cancer progression [38,53]. In the present study, we identified an interaction between PSAT1 and PKM2 and a need for nuclear PKM2 in the PSAT1-driven motility of EGFR-activated NSCLC cells.

PKM2 functions as the predominant pyruvate kinase in many tumor types and promotes growth by various mechanisms including regulating anabolic reactions [39]. For example, flux through serine biosynthesis is partially controlled through a feedback loop involving PKM2 activity [40,42]. Serine deprivation reduces PKM2 activity, resulting in the accumulation of the glycolytic intermediate 3-phosphoglycerate necessary for cellular serine biosynthesis [42]. Conversely, the restoration of intracellular serine induces PKM2 activity by allosteric binding. In line with this, small molecule activators of PKM2 predominantly exert their anti-tumorigenic effect under serine deprivation conditions in vitro [41,43]. As these findings indicate a metabolic cross-talk between PKM2 activity and serine, we speculated that the depletion of PSAT1 in cells may alter the intracellular pyruvate kinase activity of PKM2 either through reduced serine levels or potentially through the disruption of the protein–protein interaction. However, A549 and PC9 cells did not exhibit a significant change in PKM2 expression or activity in response to PSAT1 silencing, suggesting that a PSAT1 association does not influence the intracellular PKM2 activity. As our studies were performed in a serine-proficient medium, we postulate that serine import may have sustained intracellular serine levels, which may have contributed to the lack of effect on the pyruvate kinase activity in the absence of PSAT1.

Among the oncogenic drivers of a lung adenocarcinoma, activating mutations within the EGFR tyrosine kinase domains account for approximately 17% of these diagnoses [54]. As with GBM, nuclear PKM2 has been detected in EGFR-mutant NSCLC cancer cells but not in EGFR wild-type NSCLC cells [8,44,55]. While nuclear PKM2 mediates EGFR-induced proliferation, epithelial-mesenchymal transition (EMT), migration and invasion in GBM, HCC and nasopharyngeal carcinoma cells [8,56,57,58], an EGFR-mediated nuclear function of PKM2 in lung cancer remains elusive despite a finding as a predictor for a response to PARP inhibitor treatment in EGFR-mutant NSCLC cells [44]. In addition, prior work has also demonstrated that EGF stimulation promotes cell migration in A549 NSCLC cells that express wild-type EGFR [46,47]. Based on these studies, we speculate that PSAT1 might participate in cell migration under EGFR activation through facilitating the nuclear localization of PKM2. While the suppression of PSAT1 decreased the EGFR-induced nuclear localization of PKM2, nuclear PKM2 was rescued upon PSAT1 restoration or elevated under the ectopic expression of FLAG-PSAT1, implicating a requirement for PSAT1 in PKM2’s translocation. Accordingly, cell migration decreased upon PSAT1 suppression and increased when PSAT1 was overexpressed. Various signals, including EGF, can stimulate PKM2 acetylation at K433, which is required for nuclear function and contributes to the tumor progression [7,50]. Consistent with these studies, we found that the restoration of acetyl-mimetic nuclear PKM2, but not wild-type PKM2, partially reverts the migratory defect due to the suppression of PSAT1. Therefore, our results indicate a correlation between the PSAT1-mediated cell migration and nuclear localization of PKM2 upon EGFR activation.

**Figure 8:**
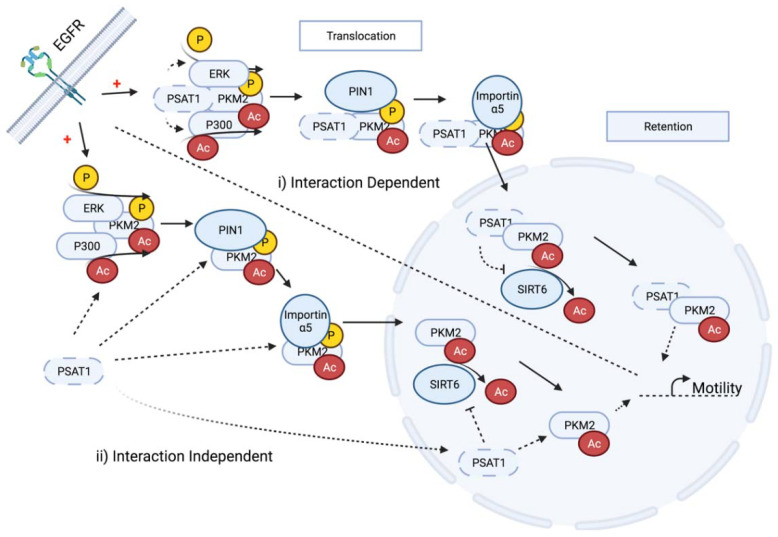
Schematic depicting putative nodes for PSAT1 regulation in the nuclear translocation and retention of PKM2 in response to EGFR activation. (**i**) PSAT1 interaction may promote cytoplasmic PKM2 phosphorylation and acetylation or suppress nuclear SIRT6-dependent deacetylation that contributes to nuclear PKM2 function in increasing pro-motility gene expression. (**ii**) PSAT1 may influence various steps involved in PKM2’s translocation and retention independent of direct PKM2 interaction. Dotted lines indicate putative PSAT1 regulatory steps.

While we have demonstrated a functional requirement for PSAT1 in an EGFR-dependent PKM2 nuclear translocation, these findings do not directly address the nuclear function of PSAT1, the requirement for the PSAT1–PKM2 interaction for a nuclear import or the effect on putative EGFR-dependent PTMs for this process, thus raising new unanswered questions. First, similar to PKM2, we also observed the EGFR-activation-dependent translocation of PSAT1 but the mechanisms that mediate PSAT1 localization still need to be elucidated, such as a putative requirement for PSAT1 modification. Second, how PSAT1 regulates PKM2’s nuclear localization is still unknown. In order to exert its nuclear function under EGFR activation, PKM2 must not only translocate but also be retained within the nuclear compartment (Figure 8). PSAT1 may function in the translocation process from cytoplasm to nucleus via the promotion of established ERK-dependent phosphorylation, PIN1-mediated isomerization and importin α5 interaction that are required for the nuclear import (Figure 8) [59]. Third, while we demonstrate a PSAT1–PKM2 association, it is still unclear whether PSAT1’s putative influence on this process is interaction-dependent or -independent or which of these individual steps may be impacted upon PSAT1 loss (Figure 8). This conceivably can be addressed by the expression of complete interaction-deficient PSAT1 or PKM2 mutants to assess their relevance on the nuclear localization of both proteins. Fourth, EGFR activation is known to induce P300-mediated PKM2 acetylation at K433 and this modified PKM2 has been observed in the nucleus [7]. Although the requirement for acetylation for PKM2’s cytoplasmic to nuclear translocation remains obscure [7,50], it is understood that K433 acetylation is necessary for its nuclear function whereas SIRT6 deacetylation leads to a PKM2 nuclear export [7,8,50] (Figure 8). Therefore, it is intriguing whether PSAT1 contributes to PKM2’s nuclear translocation or retention by regulating its acetylation status through either promoting P300 activity or suppressing nuclear SIRT6 (Figure 8); unfortunately, a lack of a commercially available PKM2 acetyl-specific antibody hindered the examination of any analysis of acetylation changes in response to PSAT1 depletion. Given the complexity of the nuclear localization and the retention of PKM2 as mentioned above, it is worth identifying which step/s is influenced by PSAT1 either in an interaction-dependent or -independent manner and these studies are the focus of ongoing work. While not depicted in Figure 8, PKM2 nuclear retention is also driven by PARP activity or the poly(A) ribose binding ability [44], which may be another means by which PSAT1 may contribute to nuclear PKM2. Together, we postulate that PSAT1 depletion may impact multiple regulatory nodes that mediate EGFR-promoted nuclear PKM2 localization and function. While it is still unclear concerning PSAT1’s requirement for regulating PKM2 acetylation, our results suggest that nuclear acetylated PKM2 is required for PSAT1-mediated cell motility. Further, as nuclear PKM2 is required for EGF-involved EMT-mediated cell migration in HCCs, oral squamous carcinomas, and colon cancers [56,60,61], further work is also needed to fully define the mechanisms by which nuclear PKM2 promotes cell motility under EGFR activation in NSCLC, potentially through mediating the transcriptional activation of migration-dependent genes (Figure 8). Finally, a limitation of this study is that these findings were primarily observed in an EGFR-mutant cell model that harbors the exon 19 deletion. The influence of other known EGFR mutations, such as the L858R or the resistance-mediated mutant T790M, were not directly examined in this work. While we use complimentary approaches involving EGF-stimulation, the inclusion of additional systems such as H3255 (L848R) or H1975 (L848R, T790M) and other EGFR-WT models (H358) would strengthen the relevance of PSAT1 in mediating the EGFR-dependent PKM2 localization and are the focus of ongoing work.

Lastly, the partial rescue observed within the PKM2 expression studies indicate that other PSAT1 activities contribute to EGFR-activated NSCLC motility that are independent of nuclear PKM2, which may include putative nuclear-specific PSAT1 function(s). As the metabolic activity of PSAT1 has not yet been thoroughly examined in this context, its enzymatic activity on cell migration and the nuclear localization of itself and PKM2 needs further investigation, potentially through the use of metabolic-deficient PSAT1 mutants such as those recently described that lack the ability of PLP binding [62].

## 5. Conclusions

We have identified PKM2 as a new PSAT1-associating protein in NSCLC cells. Whereas PSAT1 appears to be dispensable for PKM2’s pyruvate kinase activity, it is essential for PKM2 nuclear localization in EGFR-activated NSCLC cells. This supports, in part, PSAT1’s ability to promote cell migration under EGFR signaling, which may be a contributing determinant for its negative correlation with patient outcomes in EGFR-mutant NSCLC.

## Data Availability

Not applicable.

## Supplementary Materials

The following are available online at https://www.mdpi.com/article/10.3390/cancers13163938/s1, Figure S1: Genomic deletion of PSAT1 recapitulates the phenotype of shRNA-mediated PSAT1 silencing in PC9 cells. Figure S2: Ectopic expression of PSAT1 induces PKM2 nuclear localization and cell migration in EGFR-mutant PC9 cells. Figure S3: Stable PSAT1 suppression does not impact PC9 cell proliferation under medium conditions and timing used within cell migration assays. Figure S4: *PSAT1* is elevated in EGFR-mutant NSCLC compared to normal lung and correlates with poorer patient outcomes. Figure S5: PSAT1 loss abrogates nuclear PKM2 expression, which is rescued upon PSAT1 restoration. Table S1: PCR primers for PKM2 site-directed mutagenesis.
